# Slow Time-Varying Batch Process Quality Prediction Based on Batch Augmentation Analysis

**DOI:** 10.3390/s22020512

**Published:** 2022-01-10

**Authors:** Luping Zhao, Xin Huang

**Affiliations:** College of Information Science and Engineering, Northeastern University, Shenyang 110819, China; hx1970617@163.com

**Keywords:** batch process, partial least squares, batch augmentation, quality prediction

## Abstract

In this paper, focusing on the slow time-varying characteristics, a series of works have been conducted to implement an accurate quality prediction for batch processes. To deal with the time-varying characteristics along the batch direction, sliding windows can be constructed. Then, the start-up process is identified and the whole process is divided into two modes according to the steady-state identification. In the most important mode, the process data matrix, used to establish the regression model of the current batch, is expanded to involve the process data of previous batches, which is called batch augmentation. Thus, the process data of previous batches, which have an important influence on the quality of the current batch, will be identified and form a new batch augmentation matrix for modeling using the partial least squares (PLS) method. Moreover, considering the multiphase characteristic, batch augmentation analysis and modeling is conducted within each phase. Finally, the proposed method is applied to a typical batch process, the injection molding process. The quality prediction results are compared with those of the traditional quality prediction method based on PLS and the ridge regression method under the proposed batch augmentation analysis framework. The conclusion is obtained that the proposed method based on the batch augmentation analysis is superior.

## 1. Introduction

As an important mode of industrial production, batch processing is closely related to modern people’s life, and is widely used in many fields. In recent years, the fast-changing market of modern society has brought more urgent demands on products, such as multiple varieties, multiple specifications, and high quality, so that the modern process industry is more dependent on batch processes for producing small-batch and high value-added products. Therefore, the pursuit of high-quality products using batch process production has become the focus of attention.

Batch processing usually needs several minutes or even days to obtain the final quality after the end of a batch operation cycle. After various quality tests, the quality data can be collected and stored in the database. Therefore, serious time lags exist before obtaining the product quality measurement, which makes it impossible to feed back the quality information to the process control system timely during the current batch operation cycle. Thus, precise control cannot be carried out. This problem has become the bottleneck in the field of industrial quality control of batch processes. However, the other process variables of batch processes, such as pressure, temperature, flow, speed, and valve opening, can be accurately measured online. By observing and calculating the specific relationships between the process variables and the final quality, the final quality of products can be analyzed and predicted online. Therefore, how to extract high-quality information from the vast data ocean and make full use of it to guide production has attracted the attention and interest of researchers. Principal component analysis (PCA) [1,2,3,4], independent component analysis (ICA) [5,6,7,8] and partial least squares regression (PLS) [9,10,11,12], which are the core of multivariate statistical analysis technology, have been more and more favored by researchers and field engineers because they only need process data and quality data to build models, and they have unique advantages in processing high-dimensional and highly coupled data.

Compared with successive industrial processes, the process characteristics of batch production are more complex. Batch process data usually form a three-dimensional matrix. In order to effectively utilize the unique advantages of multivariate statistical methods in processing high-dimensional and highly coupled data, it is necessary to extend multivariate statistical process modeling methods to batch processes according to the data characteristics of batch processes. Multiway PCA (MPCA) and multiway PLS (MPLS) were first applied to batch processes by Nomikos and MacGregor [13,14], which led to a series of upsurges in research based on multiway statistical analysis. Many international research groups have invested a lot of manpower and material resources to carry out the research work for batch processes. The latest research applied various methods to the quality prediction and fault detection of batch processes, such as multi-mode batch process fault detection based on statistical difference LPP [15], and the fault detection of multiphase batch processes based on SVDD [16].

The above methods were originally developed to focus on the static relationship rather than the time-varying relationship between process variables. However, batch processes are essentially time-varying processes, and time-varying behaviors may exist not only in the within-batch operations but also between batches. To describe this kind of batch process, it is necessary to capture two kinds of time-varying characteristics of batch processes, that is, intra-batch time-varying, which occurs along the time direction within a batch, and inter-batch time-varying, which occurs along the batch direction through the whole process. For intra-batch time-varying, the most typical methods are based on phase division. Initially, a new phase-based sub-PCA modeling method was proposed by Lu, Gao, and Wang [17]. After phase division, a two-way PCA model was built within each phase. Thus, different phase characteristics were captured by different models and, accordingly, different process monitoring control limits were calculated for different phases. Superior monitoring results were obtained compared with the traditional MPCA. Furthermore, Zhao, and Wang [18] proposed the multiphase quality residual recursive model for the quality prediction of batch processes where the relationship between phases was analyzed to investigate the intra-batch characteristic. In their work, phases were considered to contribute to the final quality together, and each phase contributed one part of the final quality. The predicted quality residuals of the first phase are considered as the object used to predict the second phase, and this procedure is repeated phase by phase. For inter-batch time-varying, multiple modes can be divided along the batch direction and different models can be established for process analysis and quality prediction. Zhao and Gao [19] tracked the inter-batch evolution in their work. According to different batch characteristics, a few modes were separated, modeled, and monitored along the batch direction. In order to analyze the change between batches, sliding window models were established. Based on the comparison between reference windows and sliding windows, different process modes were separated in order along the batch direction. Zhao and Yuan [20] mainly analyzed the multi-mode characteristics of the injection molding process. The intermodal quality analysis was carried out to judge the relationship between the new mode and the historical mode in the modal library, and then it was determined whether the new mode was needed to update the modal library. 

Recently, the impact of the previous batches on the quality of the current batch has excited the attention of researchers. In the traditional methods, only the process data within the current batch is responsible for the quality of this batch [21]. However, it has been found that some phases of the batch before the current batch may have a greater impact on the final quality of the current batch than the phases of the current batch, which should be considered in the regression modeling. Based on this find, Zou [22] established multiple sliding window models in the batch direction for quality prediction of the injection molding process. In their work, based on the process knowledge that the plasticizing phase should have a bigger influence on the next batch than the current batch, the plasticizing phase was moved down by one batch when establishing the regression model; that is, in order to build the regression model of the current batch, the relationship between the process variables of the plasticizing phase of the previous batch and the final quality of the current batch was analyzed. Compared with the traditional method, their method improved the accuracy for quality prediction. However, obviously, this kind of movement is based on the process knowledge and the impact is arbitrary to some degree. In most cases, it may not be enough to consider moving the concerned phase by only one batch.

In this paper, focusing on the slow time-varying characteristic, a series of works have been conducted to implement an accurate quality prediction for batch processes. Based on the mechanism characteristics of batch processes, this paper puts forward the conjecture that the quality of the current batch is not only related to this batch but also related to previous batches. Therefore, in this paper, the regression method based on the batch augmentation analysis is proposed to predict the final quality of slow time-varying batch processes by extracting more process data information corresponding to the process quality. To analyze the inter-batch time-varying and include all related process data in the process regression model, sliding windows are built and the concerned process variables are extended to the process data in previous batches. Thus, the process data of the previous batches, which have an important influence on the quality of the current batch, are identified and form a new batch augmentation matrix for quality prediction. Firstly, to deal with the time-varying characteristics along the batch direction, the sliding windows are constructed to analyze several batches in the direction of the current batch, where different batches are covered by different sliding windows, and multiple continuous models are established to capture the relationships between the different process variables and quality, respectively, in order to predict the final quality. Then, the start-up process is identified and the whole process is divided into two modes according to the steady-state identification. The most important mode in this work, the process data matrix used to establish the regression model of the current batch, is expanded to involve the process data of the previous batches. The idea of a support region can be found in reference [23]. This method includes the previous data in the analysis of the current batch. However, the traditional support region is fixed and does not change with the processes’ time-varying characteristic. It is necessary to judge the process variables’ influence on the quality by a novel method based on the inter-batch time-varying characteristic analysis. Using an algorithm, all the process data from this batch and the process data from previous batches, which have an important influence on the final quality of the current batch, will be included in the regression model to obtain the correlation between the process data and the current batch quality and provide a better prediction of the quality. Moreover, considering the multiphase characteristic, the batch augmentation analysis and modeling is conducted within each phase of the process. Finally, the proposed method is applied to a typical batch process, the injection molding process. The quality prediction results are compared with those of the traditional quality prediction method based on PLS and ridge regression under the proposed framework of batch augmentation analysis. The influence on the model of faults from external sources in the batch process is also considered in this paper. *T*^2^ and *SPE* statistics are used to monitor the current model. When the test batch is faulty, an alarm is generated [24]. 

The rest of this paper consists of the following parts: First, Section 2 mainly introduces the proposed method: the PLS method, slow time-varying batch process quality prediction based on batch augmentation analysis, including the establishment of the sliding window model, start-up process identification, critical-to-quality phase and batch identification, and batch augmentation modeling. In Section 3, the method proposed in this paper is applied to a practical injection molding start-up process. The results are compared with those of the traditional methods and discussed based on the graphical results. Finally, the conclusion is drawn.

## 2. Methodology

### 2.1. Partial Least Squares Method (PLS)

Partial least squares method is a mathematical optimization technique, which finds the best function match of a group of data by minimizing the sum of squares of errors. Some absolute unknowable truth values can be obtained by the simplest method, and the sum of error squares can be minimized. The object of partial least squares is two data matrixes $X(n\times m_{x})$ with $Y(n\times m_{y})$. Partial least squares can solve the problems of collinearity [25] and insufficient samples [26] in traditional multivariate regression methods.

A partial least squares model can be shown as below

(1) $$\begin{array}{l} X=TP^{T}+E=\sum_{a=1}^{A}t_{a}p_{a}^{T}+E\\ Y=UQ^{T}+F=\sum_{a=1}^{A}u_{a}q_{a}^{T}+F \end{array}$$

The PLS model will be used in this paper to analyze the relationship between the process variables within critical-to-quality phases of critical-to-quality batches and the concerned product quality variable. 

When only one quality index is considered, the predicted quality can be calculated as

(2) $$\hat{y}=X\beta$$

where β is the regression parameter.

### 2.2. Sliding Window Model Establishment

**Figure 1:**
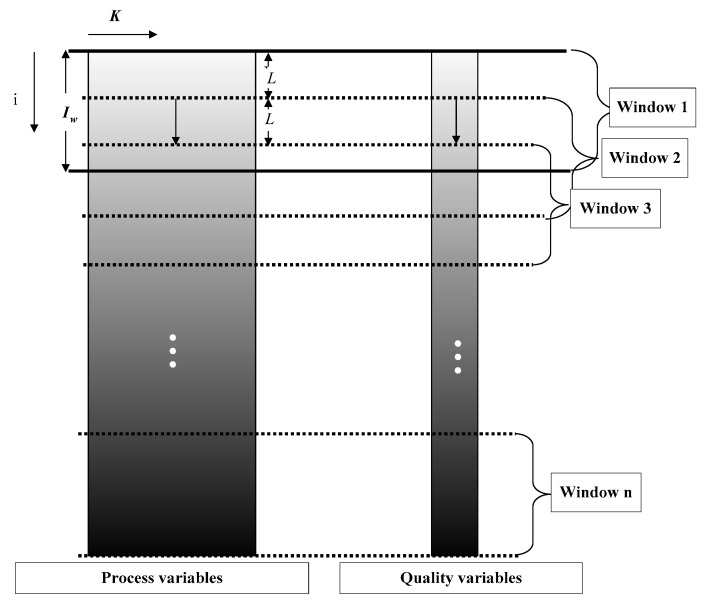
Schematic diagram of sliding window development.

The relationships between process variables and quality variables of each batch in the start-up process of one batch process are not constant but is constantly changing from batch to batch, reflecting the slow time-varying characteristic. In view of the slow time-varying characteristic, it can be approximated that the relationships between process variables and quality variables are the same in adjacent batches, while those of distant batches are different. The sliding window method is adopted to capture the slow time-varying characteristic. The relationship between the process variables and quality variables among batches covered in one window can be captured by one model, and a series of models will be built according to different windows. The development of the sliding window is shown in Figure 1. 

In the batch process, each batch has a final quality. In this paper, the width of the sliding window is set as $I_{w}$, and the window slides downward along the direction of batch by *L* batch each time. Therefore, from the first window, which has the batches 1 to $I_{w}$, to the last window, which has the batches (*I* − $I_{w}$) + 1 to *I*, a total of (*I* − $I_{w}$) + 1 windows are generated. The data size of each window is $I_{w}$ × *J* × *K*, and the corresponding quality data Y is $I_{w}$ × 1.

### 2.3. Start-Up Process Identification

Slow time-varying characteristic is obvious in the start-up process. To focus on the start-up process, it is necessary to analyze the successive batches to distinguish the start-up process from the steady process and further divide the process into modes. In this part, the stability of the final product quality is used to identify the start-up process. Steady-state identification [27,28] (SSID) method is applied to analyze the stability of the final product quality. 

Let $y_{1},\ldots ,y_{n}$ represent *n* consecutive measurement values, then the calculation formula of the variance is as follows

(3) $$s^{2}=\frac{\sum_{i=1}^{n}{\left(y_{i}-\bar{y}\right)}^{2}}{n-1}$$

where $\bar{y}$ is the average of the *n* measurements, and $s^{2}$ is the variance independently of the order of the observations. If there is a trend in an observation, then $s^{2}$ will include the effect of the trend.

Another calculation method of variance can be derived from the mean square continuous difference

(4) $$\delta^{2}=\frac{\sum_{i=1}^{n-1}{\left(y_{i+1}-y_{i}\right)}^{2}}{n-1}$$

$\delta^{2}/2$ is the calculated value of the variance to minimize the influence of trend.

Von Neumann [29] proposes that the ratio of variance $\eta =\delta^{2}/s^{2}$ of mean square continuous difference is suitable as a basis to judge whether a trend exists. What needs to be satisfied is that the data in a moving window has no change trend, and the value of Q=2/η is expected to be close to 1; on the other hand, if the data follow a curve, Q=2/η is statistically greater than 1. The critical value of trend detection can be obtained in reference [30].

### 2.4. Critical-to-Quality Phase and Batch Identification

Traditionally, critical-to-quality phases and critical-to-quality batches mean the phases and batches have an important impact on the quality of the current batch. Thus, the identification is conducted by *R*^2^, which reflects the goodness of fit of the regression model between the predicted value and the actual quality of each sampling point. *R*^2^ is used to measure the importance of quality variables to the quality variables of the quality prediction model. That is, if a prediction model has a larger *R*^2^, it means the process variables of this model have an important influence on the final quality and can be identified as critical to quality. It is easy to apply this idea to phases. That is, the phase that has the process variables that are critical to quality would be identified as critical-to-quality phase. 

To analyze the impact of previous batches on the final quality of the current batch, it is necessary to propose a strategy to conduct critical-to-quality batch identification. Thus, in this paper, within a sliding window, starting from the current batch, 1 to *N* batches are pushed forward one by one to build regression models between the moved process variables, and the final quality of the current batch and *R*^2^ values are calculated. In the same way as critical-to-quality phases, the batch which has highest *R*^2^ is identified as a critical-to-quality batch. That is, among the analysis of *N* batches, if the highest *R*^2^ is obtained when *n* batches are pushed forward, then it can be concluded that the *n*-th previous batch should be identified as the critical-to-quality batch and involved in the regression model later.

The prediction accuracy $R_{k,c,n}^{2}$ of the quality prediction model of the *k*-th sampling time within the *c*-th phase of the *n*-th previous batch is as follows

(5) $$R_{k,c,n}^{2}=\frac{\sum_{i=1}^{I}{\left({\hat{y}}_{i,k,c,n}-\bar{y}\right)}^{2}}{\sum_{i=1}^{I}{\left(y_{i}-\bar{y}\right)}^{2}}$$

where $y_{i}$ is the measured value of the quality variable of the *i*-th batch, ${\hat{y}}_{i,k,c,n}$ is the predicted value of the quality variable of the *k*-th time slice within the *c*-th phase of the *n*-th previous batch, and $\bar{y}$ is the average value of the quality variable measurement value of the *i*-th batch. The value range of $R_{k,c,n}^{2}$ is 0–1. When $R_{k,c,n}^{2}$ approaches 1, this indicates that the precision of the quality prediction model is high, so the influence of this phase on quality variables is great. On the contrary, when $R_{k,c,n}^{2}$ approaches 0, this means that the change in this phase cannot explain the change of quality index well and the influence on quality variable is small. 

When the sampling times in phase *c* are from *k_start_* to *k_end_*, $\bar{R_{c,n}^{2}}$ is proposed to indicate the final impact of the *c*-th phase of the *n*-th previous batch to the current batch

(6) $$\bar{R_{c,n}^{2}}=\sum_{k=k_{start}}^{k_{end}}R_{k,c,n}^{2}/(k_{end}-k_{start})$$

Thus, the critical-to-quality phase and batch can be determined by observing the value of $\bar{R_{c,n}^{2}}$.

### 2.5. Batch Augmentation Modeling

Based on the analysis above, the critical-to-quality phases are not limited to the current batch; that is, not only the process data of the current batch but also the process data of previous batches, which have important impact on the final quality, are identified and included in the quality prediction model, so as to obtain the meaningful information between the quality and the process data as much as possible, and, thus, the quality prediction will be improved. 

**Figure 2:**
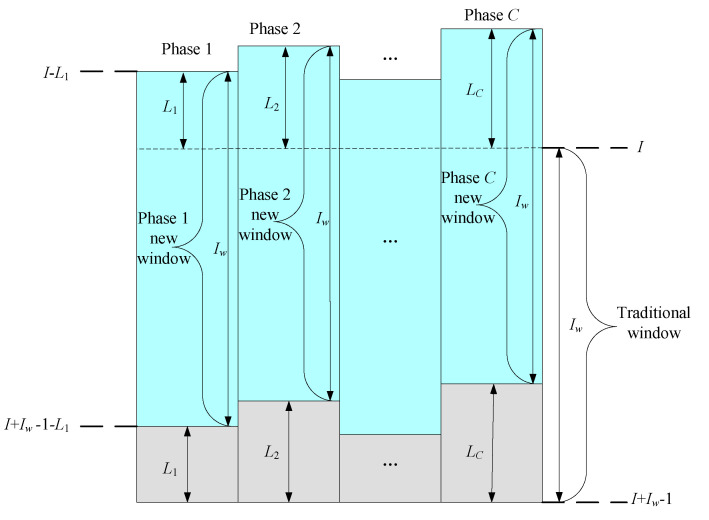
Development of sliding window with augmented batches.

In this part, for quality prediction based on inter-batch time-varying characteristic analysis, a novel method of data window construction method based on batch augmentation PLS is proposed, where the previous batches are included in the data window for quality regression of the current batch. For the applied window model, it is equivalent to augmenting *n* previous batches for the current batch analysis. For multiple phase processes, the number of augmented batches for each phase could be different according to different phase characteristics, which could be decided by the critical-to-quality phase and batch identification method proposed in last part. In this way, the development of the sliding window with augmented batches is shown in Figure 2. 

In the traditional method [21], the window constructed in each operation phase is from the *I* batch to the *I* + $I_{w}$ − 1 batch and, for all phases, the length of the window is the same, so the modeling data of phase *c* are $X_{c}$ ($I_{w}$ × *J* × *K*), *c* = 1, 2, …, *C*. While, for the proposed method, the maximum number of correlation batches of the current window, *L_c_*, is obtained according to *R*^2^ in each phase to form new modeling data, which will be different for different phases. For example, within phase 1, *L*_1_ batches are pushed forward in the window, then the start batch of phase 1 window is *I* − *L*_1_ and the end batch is *I* + $\;I_{w}$ − 1 − *L*_1_, forming the data matrix $X_{1}$ ($I_{w}$ × *J* × *K*_1_), where *K*_1_ is the number of sample points of phase 1. For phase *c*, the start batch is *I* − *L_c_* and the end batch is *I* +$I_{w}$ − 1 − *L_c_*, forming the data matrix $X_{c}$ ($I_{w}$ × *J* × *K_c_*), where *K_c_* is the number of sample points of phase *c*. Finally, the *C* phases windows are combined to form new modeling data $X_{a}$ ($I_{w}$ × *J* × *K*). Each time slice of $X_{a}$ ($I_{w}$ × *J* × *K*), $X_{k}$ ($I_{w}$ × *J*), is regressed with the quality Y to obtain the regression coefficient, and then the batch quality can be predicted by applying the PLS method.

The time slice PLS model is as below

(7) $$\begin{array}{l} X_{k}=T_{k}P_{k}{}^{T}+E_{k}=\sum_{a=1}^{A}t_{k,a}p_{k,a}^{T}+E_{k}\\ Y=U_{k}Q_{k}{}^{T}+F_{k}=\sum_{a=1}^{A}u_{k,a}q_{k,a}^{T}+F_{k} \end{array}$$

When only one quality index is considered, the predicted quality can be calculated as

(8) $${\hat{y}}_{k}=X_{k}\beta_{k}$$

where $\beta_{k}$ is the regression parameter for the *k*-th time slice, *k* = 1, 2, …, *K*.

For process monitoring, the Hotelling-*T*^2^ and *SPE* statistics for the current time *k* are

(9) $$T_{k}{}^{2}=x_{k}{}^{T}R_{k}{\left(\frac{T_{k}{}^{T}T_{k}}{I_{w}-1}\right)}^{-1}R_{k}{}^{T}x_{k}$$

(10) $$SPE_{k}={\left\|{\tilde{x}}_{k}\right\|}^{2}={\left\|(I_{J}-P_{k}R_{k}{}^{T})x_{k}\right\|}^{2}$$

where ${\tilde{x}}_{k}$ is the residual vector at the current time.

The corresponding control limits are

(11) $$\delta_{T_{k}{}^{2}}(\alpha )=\frac{H(I_{w}{}^{2}-1)}{I_{w}(I_{w}-H)}F_{k\alpha }(H,I_{w}-H)$$

(12) $$SPE_{k}(\alpha )=g_{k}\chi_{h,\alpha }^{2}$$

where $F_{k\alpha }(H,I_{w}-H)$ is the F distribution with α confidence and H and $I_{w}-H$ are the degrees of freedom, and H is the number of retained latent variables. The four-fold cross-validation method is used to determine the number of retained latent variables in this work [31,32]. $g_{k}\chi_{h,\alpha }^{2}$ is the $\chi_{}^{2}$ distribution with the same confidence level of α and the proportional coefficient of $g_{k}=s/2\mu$; $h=2\mu^{2}/s$; μ is the mean value of *SPE*; s is the variance of *SPE*.

## 3. Illustration and Discussion

### 3.1. Injection Molding Process

As the most typical batch process, the injection molding process is mainly composed of mold closing, injection, packing-holding, plasticizing, cooling, mold opening, and part ejection. The injection phase, packing-holding phase, plasticizing phase, and cooling phase are the four most important operation phases that determine the quality of parts. At the same time, the production settings in the injection molding process cause multiple production modes.

**Figure 3:**
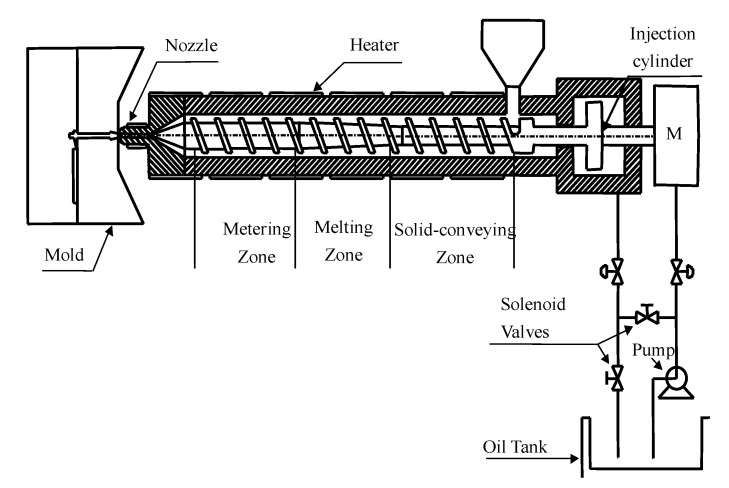
A simplified schematic diagram of injection molding machine.

A schematic diagram of the injection molding machine is shown in Figure 3. A general injection molding machine mainly comprises an injection system, a mold locking system, a hydraulic control system, and an electrical control system. The injection system has the main functions of plasticizing and fusing polymer granules or powder in the cylinder into polymer melt in the front of the cylinder, injecting the melt into the mold cavity at high pressure and high speed, and providing holding pressure in the subsequent packing-holding phase to make the polymer enter the mold cavity continuously to fill the shrinkage caused by cooling. The mold locking system has the function of opening or locking the mold by moving the movable mold plate to open or close the mold. The hydraulic system is an oil circuit supply and circulation system of the injection molding machine and provides pressure and speed loops for each actuating mechanism of the injection molding machine. The electric control system is responsible for various programs of the injection molding machine, and mainly controls various actions of the injection molding machine and various process variables of the injection molding process, including time, position, pressure, velocity, and the like.

### 3.2. Variable Analysis of Injection Molding Process and Experiment Condition

Injection molding is a complex process with multiple variables. In the process of injection molding, there are many factors that affect the quality of products, and there is a close relationship between the variables and each other. In addition, the process characteristics of the four main phases of the injection molding process are analyzed. It can be seen that various parameters in different phases have different effects on the product quality.

In the process of injection molding, temperature and pressure are two important state variables throughout the whole molding process. Their values directly determine the properties of polymer melt material and the flow behavior in the mold cavity. Therefore, in each phase of the injection molding process, temperature and pressure are important factors to determine the quality of products, and they are the preferred process variables in the process quality prediction.

In the injection phase, the screw velocity is the most important process, determining the melt flow velocity and affecting the product quality. When the mold is full, the injection cylinder continues to maintain a certain pressure. In this phase, the holding pressure is the most important process variable, which is generally achieved by controlling the pressure of the cylinder or nozzle. In the plasticizing phase, the plasticizing pressure is also an important variable. It helps to compact the material in the screw groove and empty the gas in the material. In addition, in order to better understand the material state, temperature and pressure sensors can be installed at the nozzle to directly measure the temperature and pressure of the melt in the nozzle.

In addition, the start-up process of the injection molding process has slow time-varying characteristics. Thus, the injection molding process is an ideal process to apply and verify the proposed modeling method for batch process quality prediction. Critical-to-quality process variables such as temperature, pressure, valve opening, and speed can be measured online by corresponding sensors, while quality variables can only be measured after each batch operation.

The material used in this experiment was high density polyethylene (HDPE). The weight was selected as the final prediction criterion, and the selected process variables are shown in Table 1, which are used to establish the model. The variable data shown in the table were collected by sensors and the operating conditions are shown in Table 2.

### 3.3. Start-Up Process Identification

A total of 100 batches are obtained in the injection molding process, with 6 variables and 919 sampling points in each batch. Therefore, the collected data are X (100 × 6 × 919) and Y (100 × 1).

**Figure 4:**
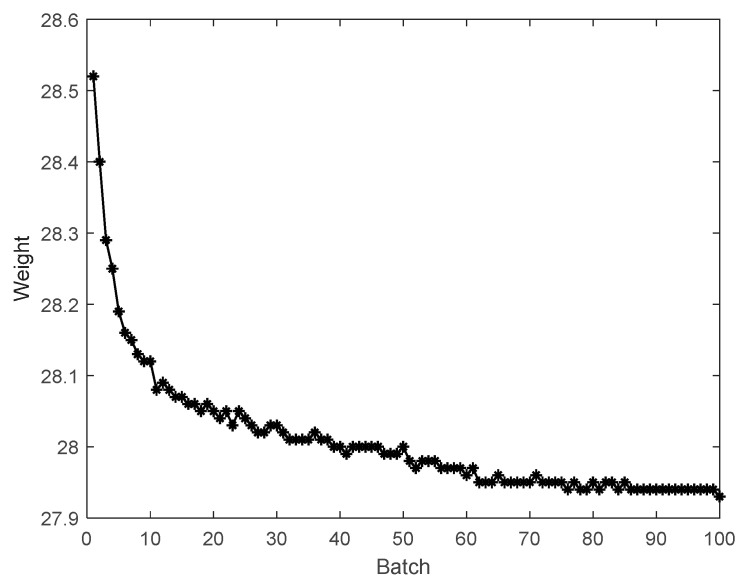
Product weights.

**Figure 5:**
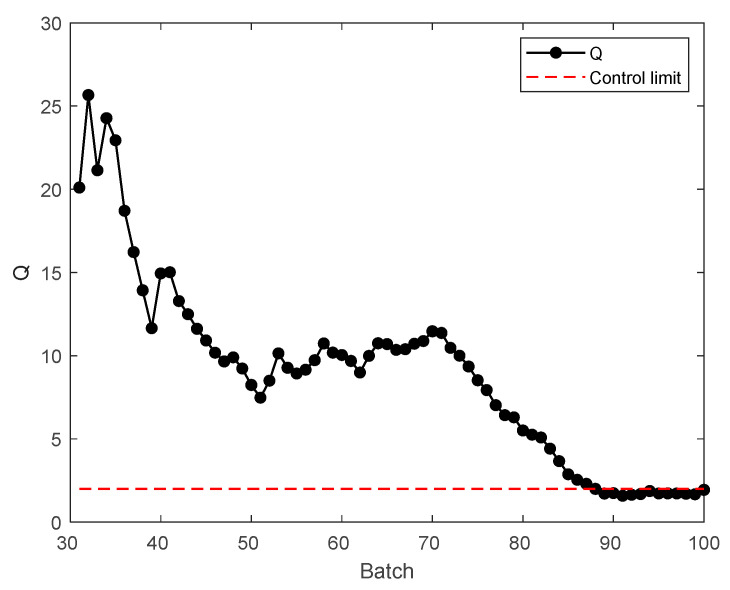
Judgment of product quality stability.

In the injection molding process, the product weights reflect the trend of the process. All weights are plotted in Figure 4. The conventional SSID method based on the ratio of variances method was used to analyze the quality change trend between batches. The analysis result is shown in Figure 5. The window length was selected to be 30. *L* was selected to be 1. The critical values for trend detection can be found in reference [29]. The confidence level of the control limit was set to 95%, and the control limit is shown by dotted line.

It can be seen from the above figure that the control limit mainly divides these batches into two parts, and the process becomes steady after batch 88. For the convenience of further explanation, the first 88 batches with Q values above the control limit are called mode 1; the 89 to 100 batches with Q values below the control limit are called mode 2. The start-up process refers to mode 1. Mode 2 can be assumed to belong to the steady process.

### 3.4. Critical-to-Quality Phase and Batch Identification

According to the change of process correlation, a batch of injection molding process is divided into four main operation phases by using a phase division method, which are the injection phase, packing-holding phase, plasticizing phase, and cooling phase. Each phase has similar process characteristics. Therefore, process characteristics are stable in the same phase. The phase division method basically corresponds to the knowledge of the batch process. For complex and unfamiliar batch processes, the phase division method can utilize process knowledge more effectively and promote the understanding of industrial process.

**Figure 6:**
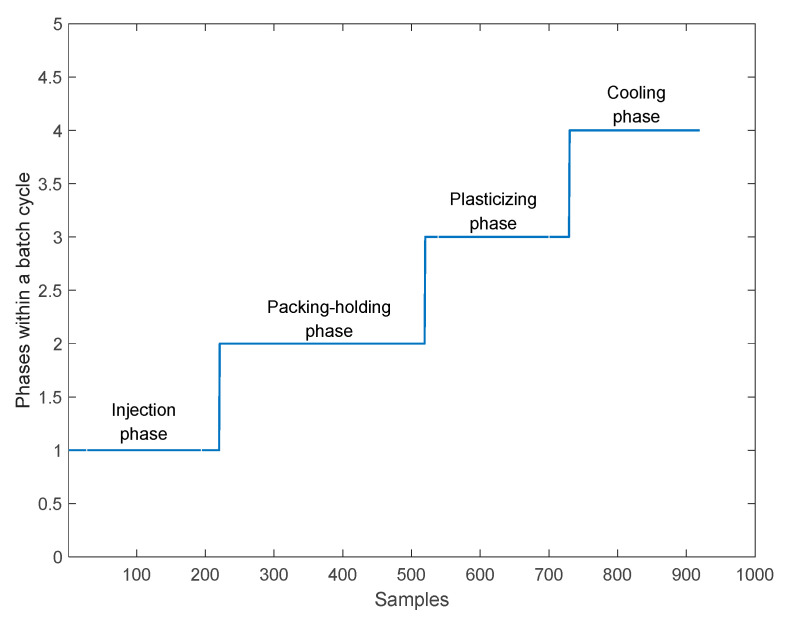
Final result of phase division in injection molding process.

The injection molding process is divided into phases by indicator variables. The phase division result of the injection molding process is shown in Figure 6. The sample points of the first phase, the injection phase, are 1–220; the sample points of the second phase, the packing-holding phase, are 221–519; the sample points of the third phase, the plasticizing phase, are 520–729; the sample points of the fourth phase, the cooling phase, are 730–919.

Since there are two different modes identified by SSID analysis, these two different modes should be considered separately. 

**Figure 7:**
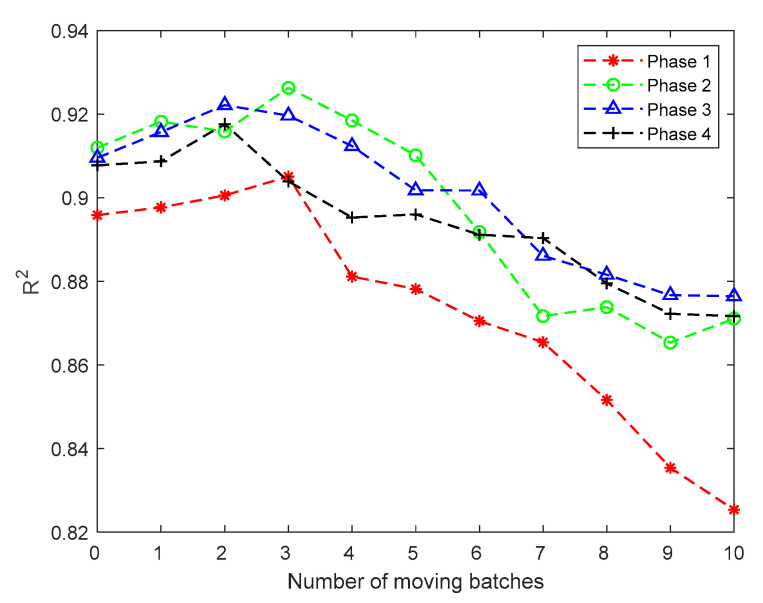
Results of mode 1.

Firstly, $\bar{R_{c,n}^{2}}$ of mode 1 is analyzed. According to the characteristics of the injection molding process, one window is selected for analysis as an example. The number of the starting batch *I* is 40, the window length $I_{w}$ is set to 30, the number of the end batch is 69, and the number of moving batches *n* is set to 10. According to the above conditions, the $\bar{R_{c,n}^{2}}$ results are shown in Figure 7. The average $\bar{R_{c,n}^{2}}$ of each batch and each phase is shown in Table 3. It can be seen from Figure 7 and Table 3 that the $\bar{R_{c,n}^{2}}$ values of each phase first increase and then decrease, and the values of $\bar{R_{c,n}^{2}}$ also achieve the maximum values between 2 and 3 batches before the current batch. It can be seen from Figure 7 that there is a maximum value of $\bar{R_{c,n}^{2}}$ when the batch is recursed forward, followed by a significant decrease. From Table 3, the batch corresponding to the maximum $\bar{R_{c,n}^{2}}$ value of each phase is selected. The following conclusions are obtained: within the first phase, three batches should be moved forward; within the second phase, three batches should be moved forward; within the third phase, two batches should be moved forward; within the fourth phase, two batches should be moved forward. According to the number of the batches that should be moved, the window for quality prediction would be rebuilt.

Since the system stabilized after 88 batches, the last window includes batches 58–87 in mode 1. All the windows in mode 1 are analyzed, and the average numbers of the batches that should be moved forward for each phase are similar among the windows, which are used for all windows in mode 1. Here, the average numbers of the batches that should be moved forward are 3, 3, 2, and 2 for the four phases.

For mode 2, which is in steady-state, because the process characteristics of the steady-state change only a little, all running batches in the steady-state are taken as a window, i.e., batches 88–100, and no other windows of steady batches are analyzed since this is not the focus of this research. 

### 3.5. Quality Analysis and Prediction of the Start-Up Process

After critical-to-quality phase and batch identification, for the quality analysis and prediction in mode 1, batch augmentation modeling is conducted for each window. The window of 40–69 batches is selected as an example for the comparative analysis, and batches 50, 55, 60, and 65, are taken as test batches. At the same time, because ridge regression is a biased estimation regression method especially used for collinearity data analysis [33,34], in this paper, under the proposed batch augmentation framework based on the process data after batch augmentation analysis, the PLS method and ridge regression method are used to predict, and predictions are compared with the traditional PLS modeling method. The advantage of the strategy proposed is analyzed and compared in this paper. In the analysis, the root mean square error (RMSE) is used to evaluate the prediction results.

Root mean square error (RMSE) can be used to test the accuracy of model prediction, which can be expressed as

(13) $$\mathrm{RMSE}=\sqrt{\sum_{i=1}^{n}d_{i}^{2}/n}$$

where *n* is the number of sampling points and *d_i_* is the difference between the actual value and the average value of the predictions. For the prediction result, the larger the RMSE is, the worse the prediction result is. Conversely, the higher the prediction accuracy is.

**Figure 8:**
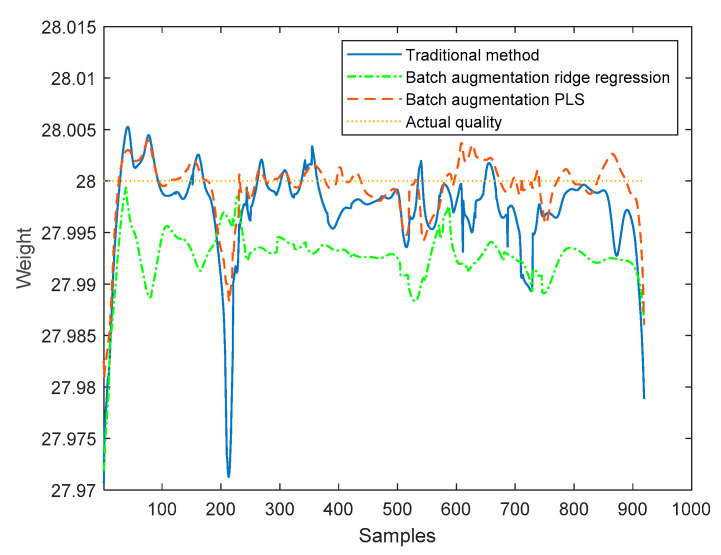
Quality prediction of batch 50.

**Figure 9:**
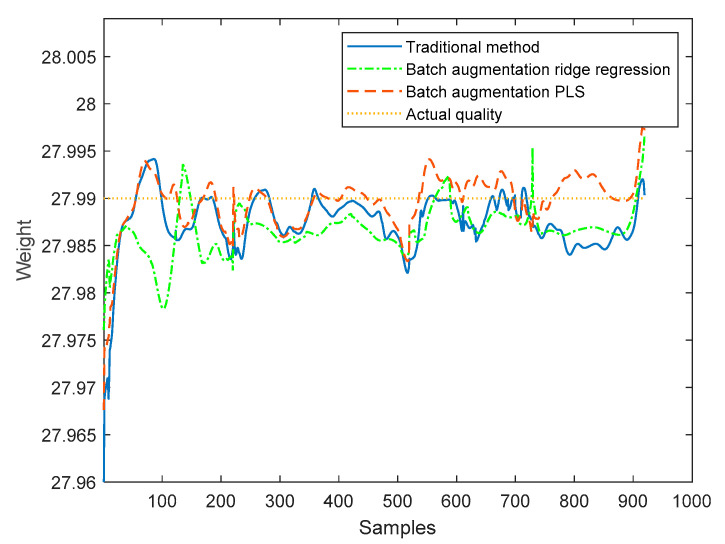
Quality prediction of batch 55.

**Figure 10:**
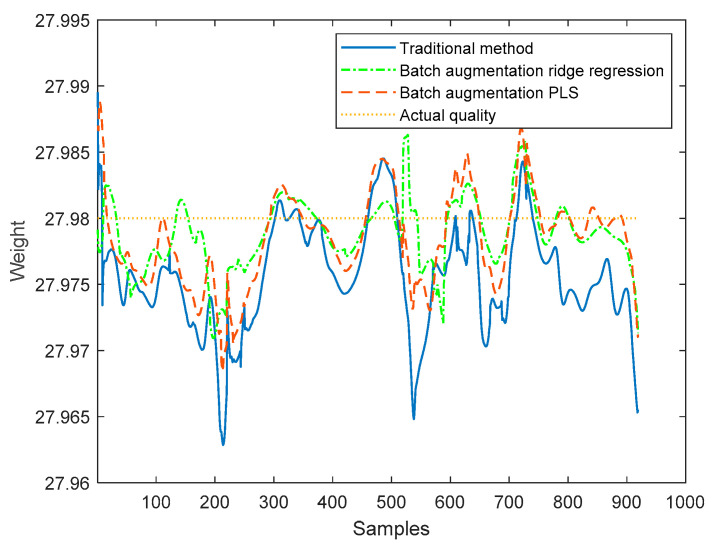
Quality prediction of batch 60.

**Figure 11:**
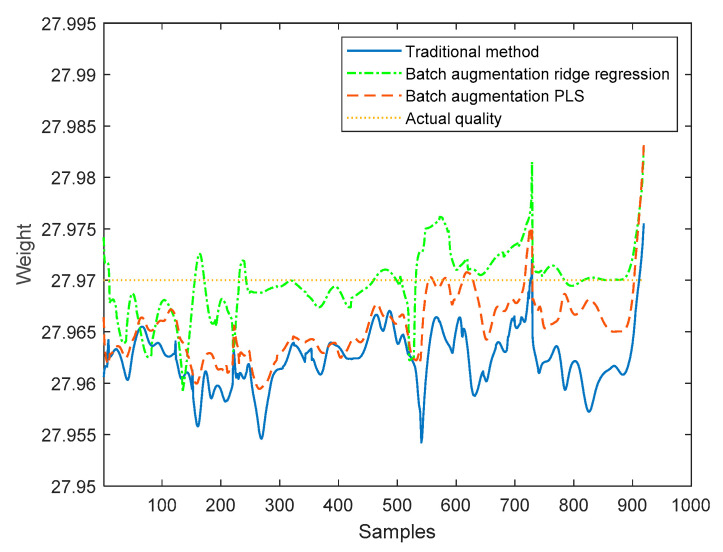
Quality prediction of batch 65.

The proposed method based on the batch augmentation analysis is used for online quality prediction of the four test batches by PLS and ridge regression, and, meanwhile, the traditional time slice PLS method without batch augmentation is used for online quality prediction of the four test batches. The online predicted quality for each test batch is shown in Figure 8, Figure 9, Figure 10 and Figure 11. It can be seen from the following four figures that the prediction results using the batch augmentation PLS are closer to the actual values than those made by the traditional method and batch augmentation ridge regression.

As can be seen from the figures, the prediction effects of the traditional method, the batch augmentation ridge regression, and the batch augmentation PLS are similar. In order to test the prediction accuracy, the RMSE is calculated based on the predicted results of each time of the test data and the final actual quality. The predicted RMSE for the four test batches are shown in Table 4. 

According to Table 4, it can be concluded that for the 50th and 55th batches, the PLS modeling method based on batch augmentation analysis has the best prediction effect, and the RMSE prediction results are 0.0032 and 0.0030, respectively. The RMSE of the traditional method are 0.0055 and 0.0041, respectively, and the RMSE of the batch augmentation ridge regression are 0.0078 and 0.0042, respectively. For the 60th and 65th batches, the ridge regression modeling method based on batch augmentation analysis has the best prediction effect, and the RMSE prediction results are 0.0029 and 0.0031, respectively. The RMSE of the traditional method are 0.0060 and 0.0082, respectively, and the RMSE of the batch augmentation PLS are 0.0038 and 0.0055, respectively.

**Figure 12:**
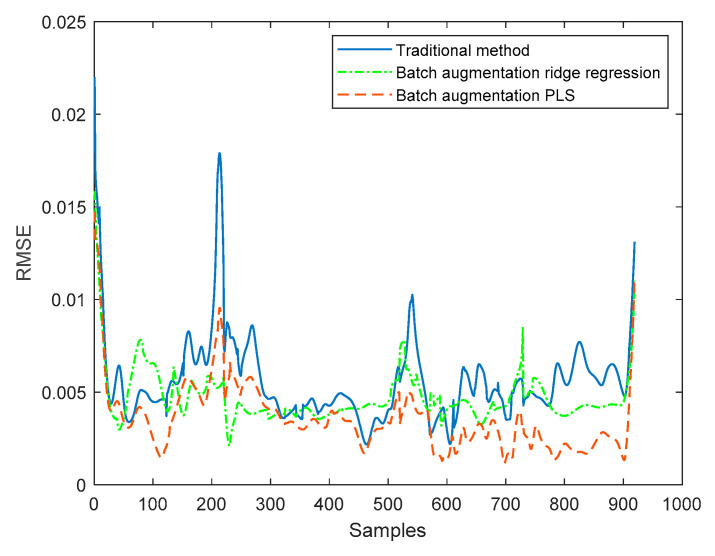
Average RMSE of online quality predictions.

Then, the online average RMSE values of the four test batches are calculated for the three methods, respectively, and the results are shown in Figure 12. The overall RMSE average values obtained are shown in Table 5. According to Table 5, the RMSE obtained by the traditional method is 0.0056, and the RMSE obtained by the batch augmentation ridge regression method is 0.0047, while the RMSE obtained by the batch augmentation PLS method is 0.0036. It can be seen that the overall RMSE of the PLS modeling method based on batch augmentation analysis is smaller than those of the batch augmentation ridge regression and the traditional method, indicating that this method has the best prediction effect.

**Figure 13:**
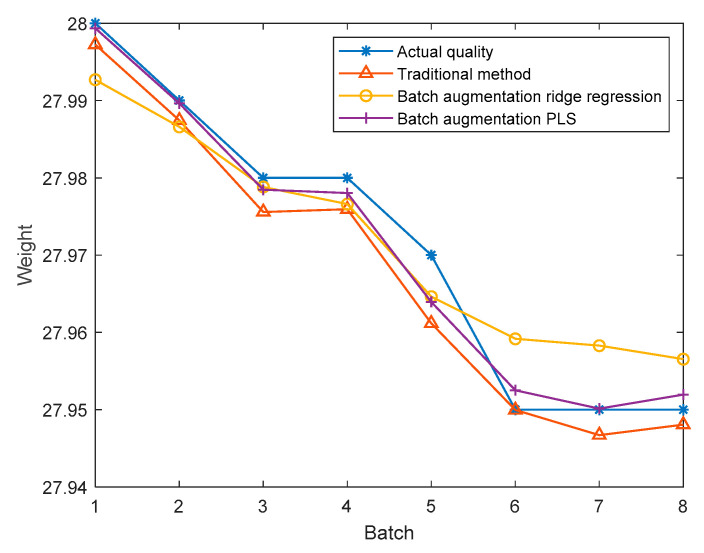
Final quality predictions.

In order to further verify the effectiveness of the method, eight test batches are randomly selected in mode 1, and the quality prediction of each test batch is carried out in the corresponding window model. The traditional method, batch augmentation PLS, and batch augmentation ridge regression method are used to predict the quality, and the average value of the predicted quality of each test batch is obtained as the final quality prediction. The results are shown in Figure 13. The RMSE is calculated from the predicted results of each batch of test data and the final actual quality. The RMSE values are shown in Table 6. According to the above table, the RMSE obtained by the traditional method is 0.0042 and the RMSE obtained by the batch augmentation ridge regression is 0.0061, while the RMSE obtained by the batch augmentation PLS is 0.0026. The RMSE of the batch augmentation PLS modeling method is the smallest. Therefore, it can be concluded that the quality prediction method based on batch augmentation PLS has higher prediction accuracy than those based on the traditional PLS and the batch augmentation ridge regression in offline state.

**Figure 14:**
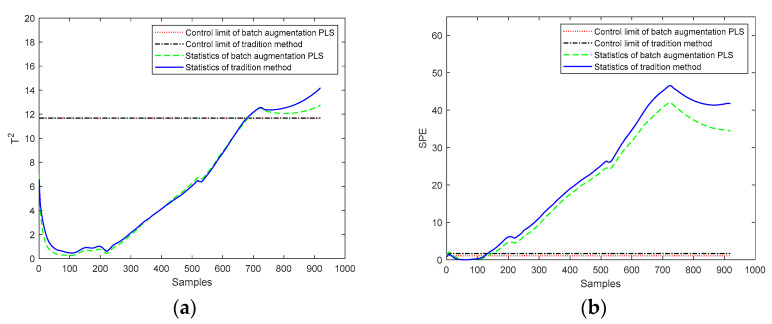
Online monitoring of the external sources’ fault. (**a**) *T*^2^ statistics monitoring, (**b**) *SPE* statistics monitoring.

In addition, there are many kinds of faults in batch processing, and the influence of faults on the model needs to be considered. For the problem of faults from external sources, the monitoring process is added to determine if a fault does not match the current model. It is necessary to identify if the process data belong to any other normal process and, if it belongs to a normal process, apply the process model to predict the quality; otherwise, a process fault is identified and an alarm should be raised. To illustrate this situation, an external input fault is artificially added to a test batch, the input temperature is increased by 5 °C after the 100th sampling point, and the current batch is monitored. The monitoring effect of the fault batch is shown in Figure 14. It can be seen from the figure that *T*^2^ and *SPE* of the fault batch are beyond the control limits. This shows that both the traditional method and the batch augmentation PLS modeling model can monitor the fault batch well. Especially for *SPE* statistics, when an error occurs at the 100th sampling point, *SPE* can quickly monitor it and generate an alarm. After testing other process models, the data do not belong to any normal process. Thus, no quality prediction will be carried out for this batch and an alarm will be raised. 

## 4. Conclusions

This paper proposes a new quality prediction method for slow time-varying batch processes: quality prediction based on batch augmentation PLS modeling and analysis. Firstly, sliding windows are adopted to capture the slow time-varying characteristics between batches. Then, according to the state characteristics, the start-up process is identified. In addition, the index of goodness of fit is used to identify the critical-to-quality phases and batches, where the previous batches are analyzed and judged for the quality analysis of the current batch. Furthermore, the batches considered to be critical-to-quality are augmented into the sliding windows, and, based on the proposed batch augmentation analysis, PLS regression models are built for quality prediction. The application to the injection molding processes showed that compared with the traditional quality prediction method and the ridge regression under the batch augmentation framework, the proposed method is the most accurate.

## Figures and Tables

**Table 1:** Process variables of injection molding process in start-up process.

Number	Variable Description	Unit
1	Screw speed	Mm/s
2	Plasticizing pressure	Bar
3	Nozzle temperature	°C
4	Cylinder pressure	Bar
5	SV2 valve opening	%
6	SV1 valve opening	%

**Table 2:** Operating condition settings for injection molding process.

Operating Parameter	Set Value
Material	High density polyethylene (HDPE)
Packing pressure	200 Bar
Packing time	3 s
Mold cooling water temperature	25 °C
Injection velocity	24 mm/s
Barrel temperature	230 °C
Cooling time	15 s

**Table 3:** Average $\bar{R_{c,n}^{2}}$ of each batch and each phase of mode 1.

Batch	Phase 1	Phase 2	Phase 3	Phase 4
*n*	0.8959	0.9120	0.9096	0.9078
*n* − 1	0.8977	0.9182	0.9157	0.9087
*n* − 2	0.9006	0.9159	0.9222	0.9176
*n* − 3	0.9051	0.9263	0.9197	0.9039
*n* − 4	0.8811	0.9185	0.9124	0.8953
*n* − 5	0.8782	0.9101	0.9018	0.8960
*n* − 6	0.8705	0.8918	0.9017	0.8912
*n* − 7	0.8654	0.8717	0.8861	0.8904
*n* − 8	0.8516	0.8738	0.8816	0.8795
*n* − 9	0.8354	0.8653	0.8767	0.8723
*n* − 10	0.8254	0.8711	0.8764	0.8717

**Table 4:** RMSE comparison of 4 test batches quality predictions.

Test Batch	Traditional Method	Batch Augmentation Ridge Regression	Batch Augmentation PLS
50 batch	0.0055	0.0078	**0.0032**
55 batch	0.0041	0.0042	**0.0030**
60 batch	0.0060	**0.0029**	0.0038
65 batch	0.0082	**0.0031**	0.0055

**Table 5:** Overall RMSE of online quality predictions.

Method	RMSE
Traditional method	0.0056
Batch augmentation ridge regression	0.0047
Batch augmentation PLS	**0.0036**

**Table 6:** RMSE of final quality predictions.

Method	RMSE
Traditional method	0.0042
Batch augmentation ridge regression	0.0061
Batch augmentation PLS	**0.0026**

## Data Availability

The data presented in this study are openly available in https://share.weiyun.com/ZdVLBo2z.
